# Dye-functionalized Sol-gel Matrix on Carbon Nanotubes for Refreshable and Flexible Gas Sensors

**DOI:** 10.1038/s41598-018-30481-y

**Published:** 2018-08-10

**Authors:** Jeongsu Kim, Haneul Yoo, Viet Anh Pham Ba, Narae Shin, Seunghun Hong

**Affiliations:** Department of Physics and Astronomy, and Institute of Applied Physics, Seoul National University, Seoul, 151-747 Republic of Korea

## Abstract

We report a colorimetric dye-functionalized sol-gel matrix on carbon nanotubes for use as a refreshable and flexible gas sensor with humidity calibration. Here, we fabricated gas sensors by functionalizing dye molecules on the top of carbon nanotube networks via a sol-gel method. Using hybrid gas sensors with different dye molecules, we could selectively detect various hazardous gases, such as NH_3_, Cl_2_ and SO_2_ gases, via optical and electrical signals. The sensors exhibited rather large conductance changes of more than 50% following exposure to gas species with concentrations even under the permissible exposure limit. Significantly, we could refresh used gas sensors by simply exposing them to fresh N_2_ gas without any heat treatment. Additionally, our sensors can be bent to form versatile practical sensor devices, such as tube-shape sensors for ventilation tubes. This work shows a simple but powerful method for building refreshable and selective gas sensors for versatile industrial and academic applications.

## Introduction

Selective gas sensors have been widely used to detect leakage of hazardous gases in living environments and industrial complexes^1–17^. One example is the colorimetric gas sensor, in which colorimetric dye molecules on a solid substrate react with specific gas species and exhibit color changes that can be used as a sensing signal^9–12^. Colorimetric gas sensors are usually very cheap and enable one to selectively detect a broad range of different gas species. However, such sensors show some disadvantages, such as a rather slow response time and a low sensitivity. Additionally, colorimetric gas sensors cannot be used to quantitatively evaluate gas concentrations. On the other hand, electrochemical gas sensors have been utilized for fast and quantitative evaluation of specific hazardous gas species^13–15^. However, electrochemical sensors are rather expensive and can only be used for a limited range of gas species. Recently, semiconducting channels based on various nanostructures have been utilized to build cheap and selective gas sensors for the sensitive and fast evaluation of hazardous gas species^1–8,16,17^. However, such nanostructure-based sensors often rely on rather *non-specific* interactions between gas molecules and nanostructure surfaces^1,2,4,8^. Thus, these sensors usually exhibit a rather poor selectivity. In addition, environmental conditions, such as humidity, can unpredictably affect the sensing signal, which makes it very difficult to use these sensors for quantitative evaluation of gas concentrations. Moreover, many of these sensors require a heat treatment to refresh used sensors for repeated measurements^16,17^.

Herein, we built a hybrid nanostructure of a colorimetric dye-functionalized sol-gel matrix on carbon nanotubes (CNT) to develop refreshable and flexible gas sensors for selective and quantitative sensing. In this sensor, the selective and reversible binding of target gas molecules onto the colorimetric dye in the sol-gel matrix caused changes in the ion density on the layer, which was measured by an underlying semiconducting carbon nanotube-based transistor. Hazardous gas species with a concentration below the permissible exposure limit caused a large conductance change in the CNT-dye hybrid gas sensor of more than 50% compared to the original conductance. This phenomenon is presumably due to the sol-gel matrix providing an ideal environment for selective gas-dye reactions and electrical signal transduction by a CNT device. To show the versatility of our strategy, we built gas sensors with different dyes to selectively detect various hazardous gases, such as SO_2_, NH_3_ and Cl_2_. Significantly, the sensor signals were recovered back to their original values without any heat treatment when the target gas was removed. Furthermore, the sensor signals exhibited a rather simple linear dependence on the environmental humidity conditions, enabling calibration of our sensor signals under varying conditions. Since our sensors provide a large signal and can be refreshed without any high temperature treatment, they are an ideal device for versatile practical applications, such as hazardous gas monitoring in public areas and industrial complexes.

## Methods

### Fabrication of CNT-dye hybrid gas sensors

**Figure 1:**
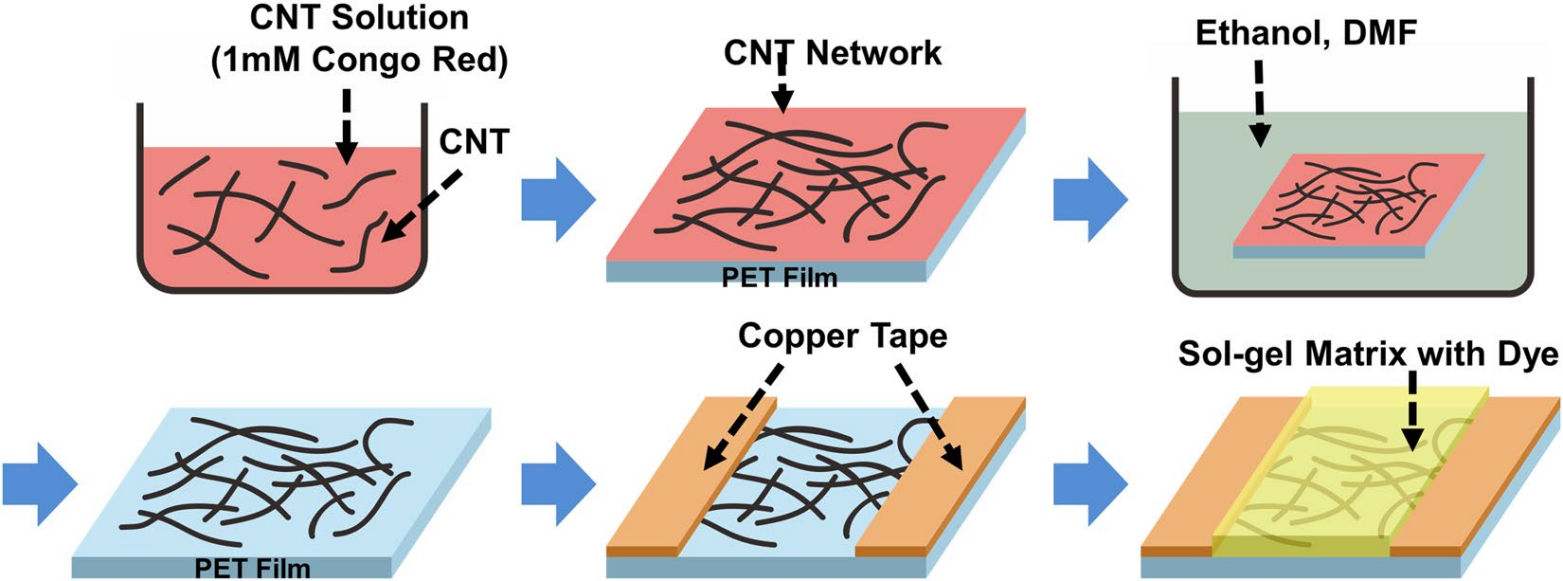
Schematic diagram showing the fabrication process of a CNT-dye hybrid gas sensor. First, a PET film was coated by a CNT network via a drop-cast method. Then, electrodes were fabricated by using copper tapes. Finally, a dye-functionalized sol-gel matrix was coated onto the CNT network and cured.

Figure 1 shows a schematic diagram depicting a fabrication method for our CNT-dye hybrid gas sensor. First, single-walled CNTs (Sigma Aldrich Inc., (6; 5) chirality, ~93% semiconducting) were dispersed in a 1 mM Congo red dye solution via a sonication using a tip type ultrasonicator for 1 hour (0.005 mg/mL). Secondly, 100 μL of the dispersed CNT solution with the Congo red dye was drop-casted onto a PET substrate. Here, Congo red dye was utilized to enhance the dispersion of CNTs in aqueous solution for drop casting CNTs on solid substrates^18^. Also, previous works show that the dye molecules on the drop-cased CNTs can be removed easily by organic solvent such as dimethylformamide. Thirdly, the PET substrate was heated on a hotplate to 100 °C for 30 min to dry out the CNT solution. Here, the CNTs were adsorbed onto the PET substrate without the coffee ring effect. Fourth, the PET substrate was rinsed with ethanol and N,N-Dimethylformamide for 30 min, respectively. Here, the Congo red molecules were removed, and only the pure CNTs remained on the PET film^18^. The removal of Congo red dye molecules was confirmed by the optical absorbance data (See Fig. S1 in supplementary data). Note that, since the Congo red dye was used only as a dispersant for CNT solution and removed from the cased CNTs on the solid substrates, it did not participate in the operation of our gas sensors. Fifth, copper tapes with a conductive adhesive were attached onto the PET substrate as electrodes. Finally, a dye-functionalized sol-gel matrix was coated onto the PET film via a previously-reported method^9^. In this method, the sol-gel matrix (Sigma Aldrich Inc.) was mixed with gas-specific dye such as methyl red or chlorophenol red, and it coated on the CNT films to functionalize the CNTs for a specific reaction with gas molecules^19^. Here, the dye molecules in the sol-gel matrix react with only a specific gas, altering the conductance of the CNTs on the substrates. Using this fabrication process, we successfully fabricated CNT-dye hybrid gas sensors without using lithography or vacuum processes. Note that, our gas sensors were composed of flexible materials; thus, the gas sensors can be bent to form versatile practical sensor devices.

### Gas detection experiment

Gas detection experiments in this paper were performed using a homemade gas chamber. The detailed structure of the gas chamber is described in the supplementary data (Fig. S2). In brief, we placed our gas sensors into a homemade gas flow cell and passed a target gas into the cell using a digital mass flow controller for gas detection experiments. Here, we mixed the target gas with a mixture of dry (0% relative humidity) and wet (100% relative humidity) N_2_ to control the concentration and humidity before injecting the gas into the cell. During the experiment, the relative humidity of the gas was monitored by a thermo-hygrometer placed in the cell. Then, changes in the conductance of our sensors due to different concentrations of gas flow were measured by a semiconductor analyzer (Keithley, 4200-SCS) with a DC bias condition and used as sensing signals. During the sensing experiment, the temperature inside the flow cell was maintained at a room temperature of 23 °C ± 3 °C.

## Result and Discussion

**Figure 2:**
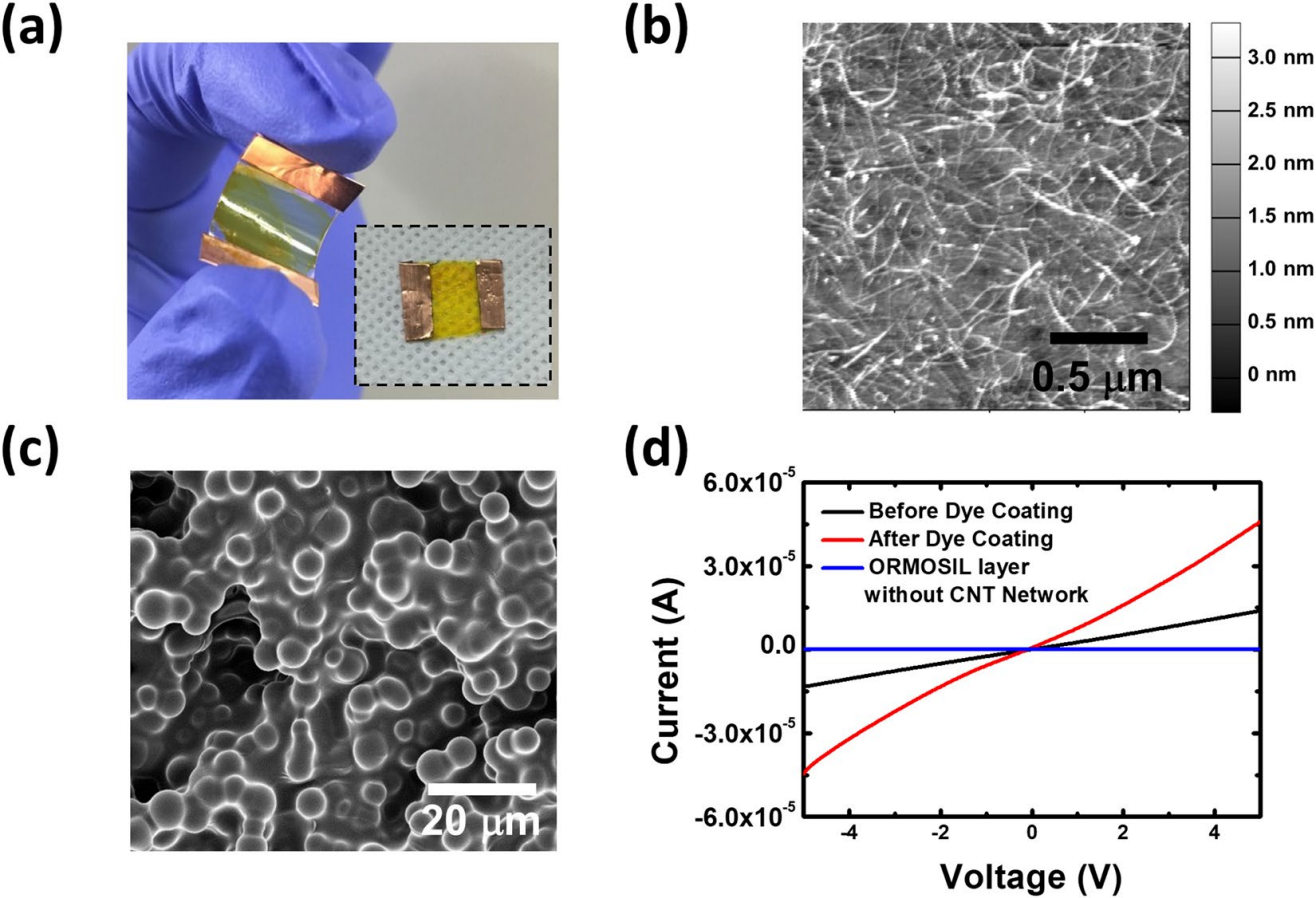
Basic properties of the CNT-dye hybrid gas sensors. (**a**) Optical image of a CNT-dye hybrid gas sensor. Here, methyl red was used for the functionalization of the sol-gel matrix on the CNT channel. (**b**) AFM topography image of CNT networks on a PET film. The gray scale bar represents the height in the image. (**c**) SEM image of the surface of a sol-gel matrix. (**d**) I–V curve of a CNT-dye hybrid gas sensor before and after the sol-gel matrix coating.

Figure 2(a) shows an optical image of a CNT-dye hybrid gas sensor. Here, we utilized methyl red dye molecules to functionalize a sol-gel matrix, and the CNT channel of the sensor was coated by the sol-gel matrix (yellow region). The size of the sensor is 2 cm × 1.5 cm. Since we utilized PET polymer films as a substrate, the as-fabricated gas sensors are highly flexible.

Figure 2(b) shows the AFM image of a CNT network on a PET film after the removal of Congo red dye solution used for CNT dispersion. The diameters of *individual CNTs* and *CNT bundles* were *approximately* 0.7~1.0 *nm* and *over* 5 *nm*, respectively. Note that, we utilized small-diameter semiconducting single-walled CNTs to fabricate the CNT transistors^20^. The AFM image shows that the CNTs are uniformly coated onto the PET surface without forming bundles. This indicates that we successfully assembled CNTs on the PET film.

Figure 2(c) shows an SEM image of a sol-gel matrix surface. The sol-gel matrix shows a highly porous structure with a rather uniform silica gel size. Since the thickness of the sol-gel matrix is approximately a few hundred micrometers, individual CNTs below the sol-gel could not be resolved in the SEM image. This porous structure can hold dye molecules and allows the transmission of target gas molecules into our gas sensors, enabling sensitive detection of gas molecules^21,22^.

Figure 2(d) shows the I–V curves of a CNT-dye hybrid gas sensor before and after coating with the sol-gel matrix. The I–V curves were measured by a semiconductor characterization system (Keithley, 4200-SCS). The bias voltage was varied from −5 V to 5 V. The I–V curves show non-linear behavior^23^. The conductivity of the sensor measured after coating with the sol-gel matrix was increased by approximately 2.67 times at 5 V. To confirm that the sol-gel matrix layer is insulating, we fabricated a gas sensor without a CNT network and found a negligible conductance (the blue line in Fig. 2(d)). This result indicates that the sol-gel matrix was insulating, while the coating of the matrix on CNTs increased the conductivity of the CNT networks. Presumably, this phenomenon is due to the sol-gel matrix on the CNT transistor changing the pH value of the environment surrounding the CNT network. It was previously reported that a change of pH value could affect the conductivity of CNT networks.

**Figure 3:**
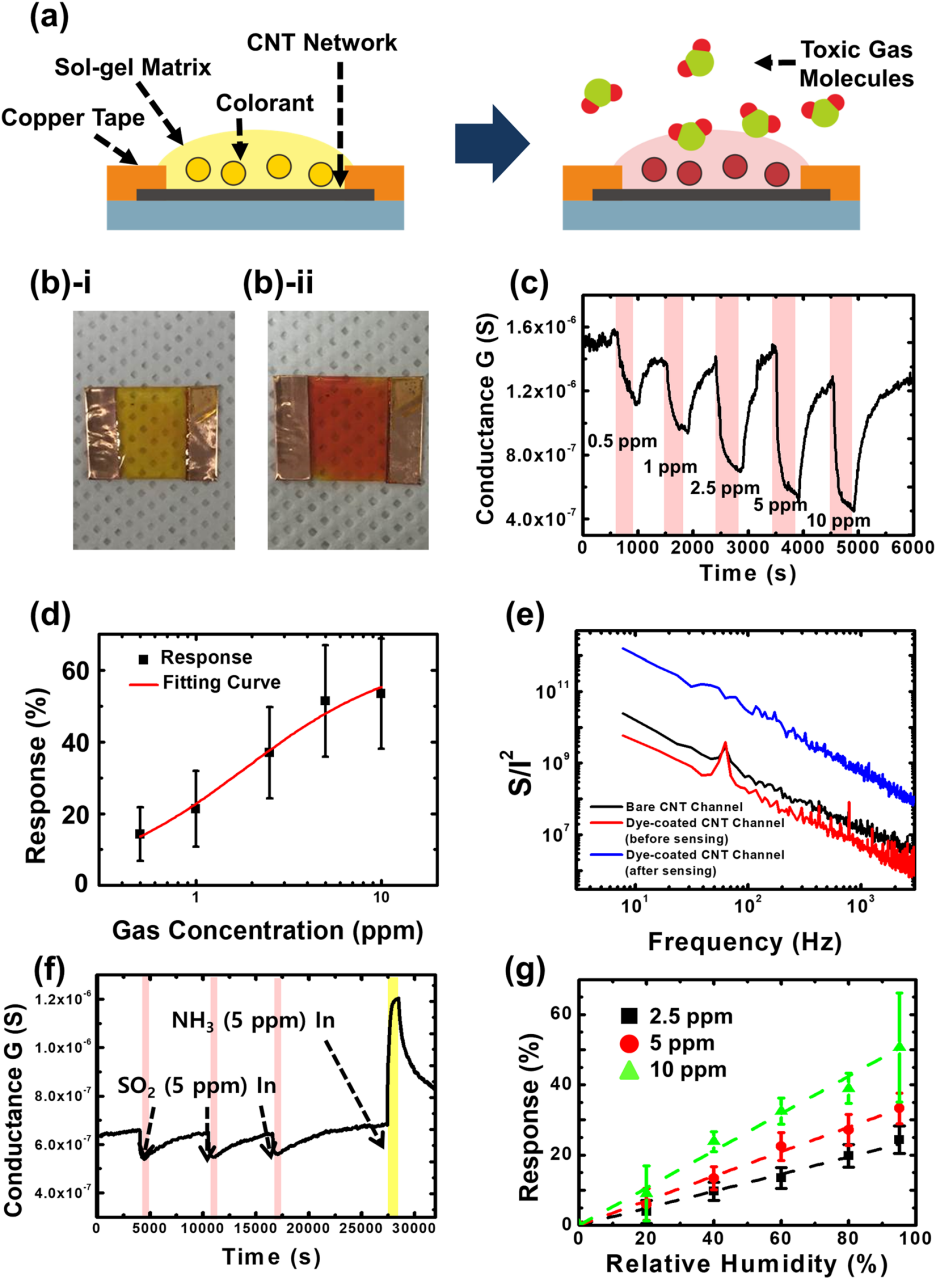
Selective and refreshable gas sensing with humidity calibration. (**a**) Sensing mechanism of a CNT-dye hybrid gas sensor. (**b**)-i Optical image of the CNT-dye hybrid gas sensor before gas exposure. (**b**)-ii Optical image of the CNT-dye hybrid gas sensor after SO_2_ gas exposure. (**c**) Real-time conductance measurement data obtained from a methyl red-functionalized CNT-dye hybrid gas sensor after the introduction of SO_2_ gases with varying concentration. (**d**) Sensor response (*ΔG*/*G*_0_) of a CNT-dye hybrid gas sensor to various concentrations of SO_2_ gas. More than 5 samples were measured for each data point. (**e**) Noise characteristics for the bare CNT networks and CNT-dye hybrid gas sensor before and after SO_2_ gas exposure. (**f**) Real-time conductance measurement data obtained for a CNT-dye hybrid gas sensor after the introduction of SO_2_ and NH_3_ gases. (**g**) Sensor response (*ΔG*/*G*_0_) of a CNT-dye hybrid gas sensor under varying relative humidity conditions. More than 7 samples were measured for each data point.

Figure 3(a) shows a plausible sensing mechanism for our CNT-dye hybrid gas sensor. Bare CNTs under ambient conditions are known to behave as a *p-type* semiconducting channel with *holes* as a majority carrier. And gas molecules adsorbed onto the CNT surface can affect the conductance of the CNT channels differently depending on its electronegativity^24^. In our sensors, the CNT channels were functionalized with *dye molecules* in a sol-gel matrix layer with some adsorbed moistures due to its porous structures. For example, methyl red dye molecules have carboxylic acid groups and phenyl rings which have a rather strong affinity to CNT surfaces^25^. Thus, the methyl red dye molecules in sol-gel matrix layer can bind to the CNT surfaces and functionalize the CNT channels. When the sensor including sol-gel matrix layer methyl red dyes was exposed to gas molecules, some gas molecules were dissolved in the sol-gel matrix layer and altered the charge states of the dye molecules, which can affect the conductance of the CNT channels^26,27^. Previous reports show that when gas molecules with a *high* (*acidic*) or *low* (*basic*) electronegativity were dissolved in water or sol-gel matrix with dye molecules, the dye molecules were charged rather *negatively* or *positively*, respectively. Eventually, the *negatively-* or *positively*-charged dye molecules on CNT channels can *capture* or *release* hole carriers, resulting in the *decreased* or *increased* conductance of the CNT channels. Thus, depending on gas molecules, the conductance change of *dye-functionalized* CNT channels can be very different.

Figure 3(b)-i,ii show optical images of our hybrid gas sensor before and after SO_2_ gas exposure, respectively. The size of the sample was 2 cm × 1.5 cm. Here, methyl red molecules were utilized to functionalize a sol-gel matrix. The concentration and exposure time of the SO_2_ gas was 5 *ppm* and 30 *min*, respectively. The relative humidity in the gas chamber was 95% during the gas exposure. The color of the sensor channel changed from yellow to red after gas exposure, indicating selective activation of the methyl red colorimetric dye molecules even in the sol-gel matrix on the CNTs. This result is consistent with the previous report about the responses of the dye molecules in water^10^.

Figure 3(c) shows the real-time change of the sensor conductance *G* during exposure to SO_2_ gas with varying concentrations. The applied bias was 0.1 V, and the relative humidity in the gas chamber was 95%. The concentration of SO_2_ gas was varied from 0.5 ppm to 10 ppm. The conductance of the gas sensor decreased following exposure to the SO_2_ gas and was recovered after exposure to N_2_ gas. Moreover, the conductance variation increased when the concentration of the applied gas was increased. This result indicates that we can estimate the concentration of the applied gas via an electrical signal. Moreover, our gas sensor showed a large conductance change of over 50% when 5 ppm SO_2_ gas (permissible exposure limit of SO_2_ gas) was applied. The similar response was also obtained in air environments (Fig. S3 in the supplementary data)^28,29^. The responses of our sensors can be explained by the specific reaction between gas and dye molecules on the CNT channels. When the methyl red-functionalized CNT channels in our sensor were exposed to SO_2_ gas molecules with a rather high electronegativity and solubility, SO_2_ gas molecules dissolved in the sol-gel matrix produced bisulfite ions and H^+^ ions^30^. Then, the H^+^ ion interacted with the negatively charged methyl red molecules near the CNT channel, which caused a decrease in conductance. It should be mentioned that this response of our dye-functionalized CNT channels to SO_2_ gas was actually opposite to that of bare CNT channels reported previously^31^. Moreover, due to the porous structure of the sol-gel, the produced bisulfite ion was rapidly changed to an SO_2_ gas molecule when the flowing SO_2_ gas was changed to N_2_ gas. This result led to the recovery of the conductance of our sensor^21,22^. The rather quick recovery of our sensors when exposed to N_2_ gas support that SO_2_ gas did not produce strong acid like sulfuric acid and did not degrade the sensor chemically. The interaction between SO_2_ gas molecules and dye molecules is relying on an acid-base interaction, and it is much stronger than the Van der Waals-type interactions between bare CNT surfaces and gas molecules in previous CNT-based gas sensors, resulting in a rather large sensor signals^9,10^. Also, a sol-gel matrix in our sensors can provide ions like liquid environments, which could provide an ideal environment for the gas-dye interactions and the following gating effects on the CNT transistor for a large electrical signal transduction.

Figure 3(d) shows the averaged sensor response, which is defined as *ΔG*/*G*_0_, for the CNT-dye hybrid gas sensors at various SO_2_ gas concentrations. We repeated the gas sensing for more than five gas sensors to estimate average values and standard deviations. These sensor response data can be analyzed by the Hill equation (red solid line in Fig. 3(c))^32^. Here, we assumed that the sensor response (*ΔG*/*G*_0_) is approximately linearly proportional to the surface density Cs of gas molecules adsorbed on the dye molecules. Then, the sensor response can be written as

1 $$\frac{{\rm{\Delta }}{\rm{s}}}{{G}_{0}}\sim k{C}_{s}=k\frac{{C}_{s,max}\cdot {C}^{n}}{{K}_{d}^{n}+{C}^{n}}$$

where *k, C*_*s,max*_*, n, C* and *K*_*d*_ are the *sensor response coefficient, maximum surface gas density, Hill coefficient, concentration* of SO_2_ gas and *dissociation constant*, respectively. From the fitting curve, the *Hill coefficient* and *dissociation constant* were estimated as 1.028 and 0.919 × 10^−7^ M, respectively. The estimated dissociation constant is similar to the value reported for the case when gas molecules bind to dye molecules in water at 25 °C^33^. We can also estimate the theoretical dissociation constant in our CNT-dye hybrid gas sensor structures. The detailed chemical reaction process and calculation process are given in the supplementary data (Equations S1–S5). In brief, the dissociation constant in our device structures was estimated by multiplying the dissociation constant for the SO_2_ acid hydrolysis reaction and the dissociation constant for the methyl red dye. The estimated Hill coefficient is close to 1, indicating that the affinity of the SO_2_ molecules for the binding sites was not dependent on other SO_2_ molecules bound on the sensor. These results show that our sensors can be used for reliable quantitative analysis of gas species based on the sensor signals.

Figure 3(e) shows the noise characteristics of a bare CNT channel and a sol-gel matrix-coated CNT channel (before and after exposure to 10 ppm SO_2_). The noise characteristics were measured using a fast Fourier transform network analyzer (SR 770). A bias voltage of 4 V was applied to the gas sensor for all noise measurements. The bare CNT network and our sensors exhibited a typical 1/*f* noise behavior^34,35^. Interestingly, the noise power density of the gas sensor was significantly increased after gas sensing, indicating that the sol-gel matrix, when exposed to SO_2_ gas, can act as a noise source. Presumably, when the SO_2_ gas binds to dye molecules, the number of positive ions in the sol-gel matrix is increased, and such positive ions can work as charge traps in the channel^36^.

Figure 3(f) shows the real-time response of our sensors to SO_2_ and NH_3_ gases. The sensing graph was measured at a bias voltage of 0.1 V and a relative humidity of 95%. SO_2_ gas (5 ppm) was applied for a duration of 500 seconds at intervals of approximately 6500 seconds (pink region). Subsequently, NH_3_ gas (5 ppm) was applied for a duration of 500 seconds (yellow region). Note that the conductance of the gas sensor decreased during repeated exposure to the SO_2_ gas. Furthermore, we could immediately apply our sensor to detect NH_3_ gas. It should be noted that all of these conductance responses were actually opposite to that observed for bare CNT channels, indicating that our sensors responded through the dye-gas molecular interactions even during repeated sensing experiments. These results clearly show that our gas sensor is reusable without any heat treatment.

Figure 3(g) shows the response of our gas sensors at different relative humidity conditions. Here, the relative humidity was varied from 0% to 95%. The concentration of the SO_2_ gas used for exposure was 2.5, 5 and 10 ppm, with more than 7 gas sensors measured for each data point. The gas sensors exhibited no response at zero relative humidity, and the response linearly increased as the relative humidity increased. Previous studies using electrochemical sensors showed that when the relative humidity is increased, the thickness of the water layer on the sol-gel matrix is also increased, and as a result, the amount of dissolved SO_2_ gas and resulting sensor signals are also increased^9,10,37^. Note that some CNT-based gas sensors in previous studies showed a linear humidity dependence^38,39^. Such a linear humidity dependence can be a significant advantage for our sensors, which enables a rather simple calibration of the sensing signal for quantitative measurement of gas concentrations. For an example, the response of the gas sensors at 10 ppm SO_2_ gas was given by

2 $$\frac{{\rm{\Delta }}{\rm{g}}}{{G}_{0}}=0.53\cdot RH\,(0\,\leqq \,RH\,\leqq \,1)$$

where *RH* is the relative humidity. We also measured the humidity dependence of the sensor response to NH_3_ and Cl_2_ gases and found a similar proportional relationship between the relative humidity and sensor response (Fig. S5 in supplementary data).

**Figure 4:**
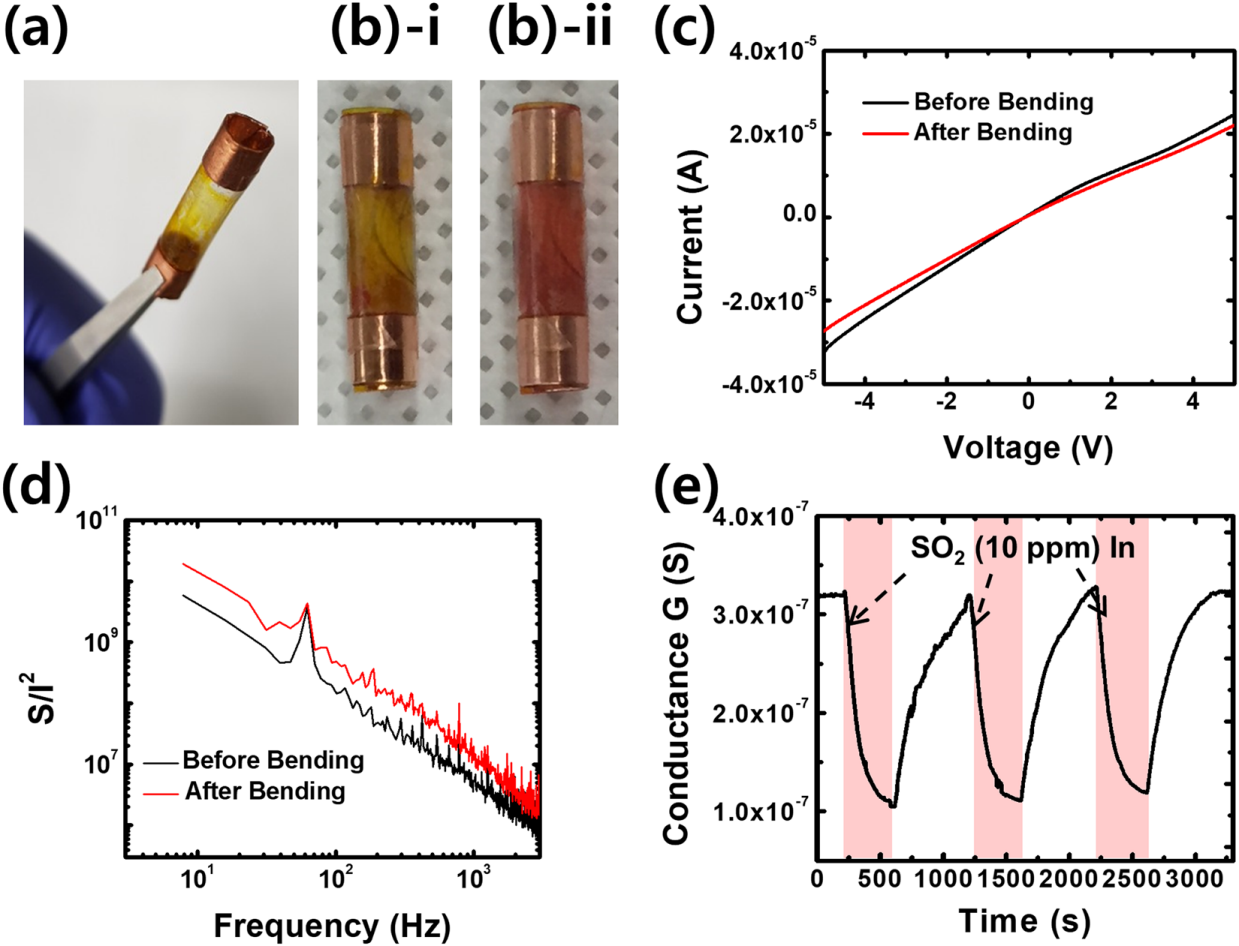
Tube-shaped gas sensors based on folded CNT sensors. We utilized methyl red-functionalized gas sensors. (**a**) Optical image of a tube-shaped CNT-dye hybrid gas sensor. (**b**)-i Optical image of a tube-shaped CNT-dye hybrid gas sensor before SO_2_ gas exposure. (**b**)-ii Optical image of a tube-shaped CNT-dye hybrid gas sensor after SO_2_ gas exposure. The color of the sensor channel was clearly changed from yellow to red. (**c**) I–V curve of the CNT-dye hybrid gas sensor measured before and during bending. (**d**) Noise characteristics of the CNT-dye hybrid gas sensor before and during bending. (**e**) Real-time conductance measurement data obtained for the bent CNT-dye hybrid gas sensor after the introduction of SO_2_ (10 ppm).

Since our gas sensors were fabricated using only flexible materials such as PET film, copper tapes and CNTs, we could easily bend the sensors to build tube-shaped gas sensors (Fig. 4(a)). The *height*, *channel length* and *radius* of the gas sensor are 20, 10 and 2.5 mm, respectively. Figure 4(b)-i,ii show photographic images of a tube-shaped CNT-dye hybrid gas sensor *before* and *after* exposure to SO_2_ (10 ppm), respectively. The color of the sensor channel changed from yellow to red, indicating that the dye molecules in the sol-gel matrix still maintained their activity even under a highly bent condition.

Figure 4(c) and (d) show the I–V curves and noise characteristics of a CNT-dye hybrid gas sensor before and after bending. The noise characteristics were measured using a fast Fourier transform network analyzer (SR 770). A bias voltage of 4 V was applied to the gas sensor during the noise measurement. Note that the I–V characteristics of the CNT-dye hybrid gas sensor did not change much even after the sensor was bent into a tube shape. The noise characteristics of the gas sensor exhibited a typical 1/*f* behavior, and they did not change much when the sensor was bent. This implies that our sensors can operate under a highly bent condition.

Figure 4(e) shows real-time electrical measurement of SO_2_ gas using a tube-shaped CNT-dye hybrid gas sensor. An applied bias of 0.1 V was used, with the relative humidity maintained at 95% during the measurement. The SO_2_ gas (10 ppm) was applied for 400 s at intervals of 1000 s. The conductance of the gas sensor decreased by 70% following exposure to gas and was recovered when the gas chamber was vented in each process. This result implies that our tube-shaped sensors operated properly and that our flexible gas sensors can be used for versatile applications requiring highly bent devices.

## Conclusions

We have developed a highly flexible and refreshable CNT-dye hybrid gas sensor for selective and sensitive gas sensing. The CNT-dye hybrid structure was fabricated by coating a colorimetric dye-functionalized sol-gel matrix layer onto a CNT network. The CNT-dye hybrid gas sensor with different dye species could be used to detect different gas species, such as SO_2_, NH_3_ and Cl_2_, with high sensitivity and selectivity. Additionally, the conductance of the CNT-dye hybrid gas sensors was easily recovered back to the original value following removal of the target gas molecules without any heat treatment. The response of the gas sensor exhibited a linear dependence on relative humidity, enabling easy calibration of our sensor for quantitative analysis of gas species. Furthermore, our sensors can be easily bent into different shapes for versatile device applications, such as tube-shaped gas sensors. This work provides a simple but quite versatile strategy for building highly flexible and refreshable gas sensors that can be utilized for various practical applications, such as monitoring hazardous gas species in industrial complexes.

## Electronic supplementary material

supplementary data
