# Supplementary material for: Dye-functionalized Sol-gel Matrix on Carbon Nanotubes for Refreshable and Flexible Gas Sensors
Source: Sci Rep. 2018 Aug 10;8:11958. doi: 10.1038/s41598-018-30481-y (PMC6086896; doi:10.1038/s41598-018-30481-y)
Supplement: Supplementary file 1 — supplementary data [file 41598_2018_30481_MOESM1_ESM.docx]

**Dye-functionalized Sol-gel Matrix on Carbon Nanotubes for Refreshable and Flexible Gas Sensors**

Jeongsu Kim^a^, Haneul Yoo^a^_,_ Viet Anh Pham Ba^a^, Narae Shin^a^ and Seunghun Hong^a, *^

^a^Department of Physics and Astronomy, and Institute of Applied Physics, Seoul National University, Seoul 151-747, Republic of Korea

^*^electronic mail: senunghun@snu.ac.kr

**Optical Absorbance of different CNT Networks on a PET Substrate**


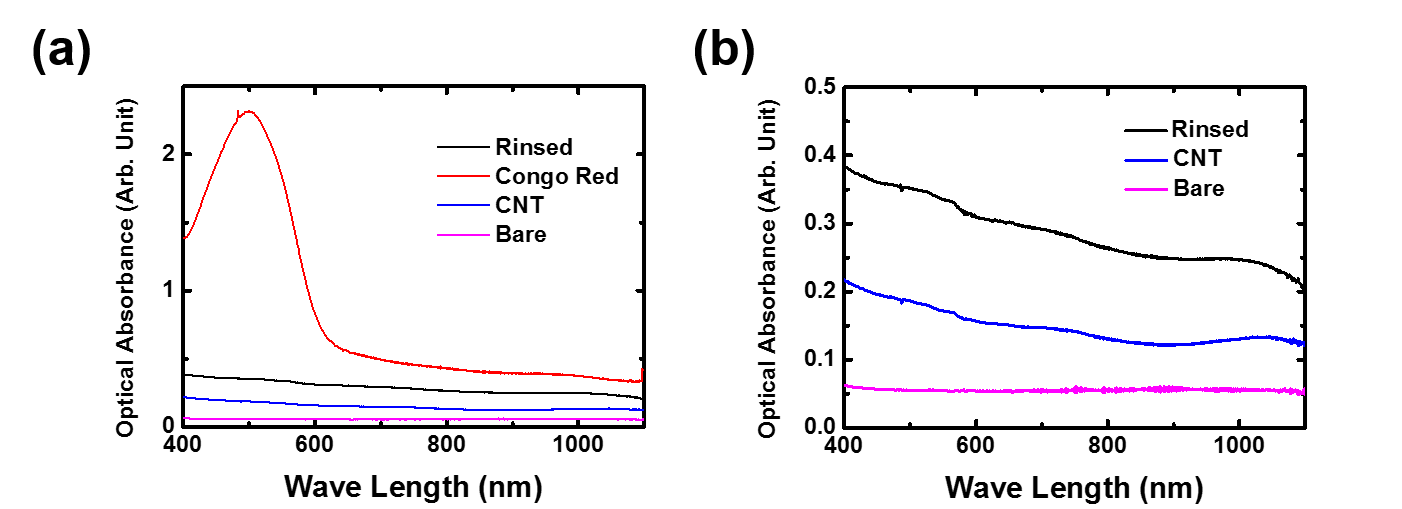


**Figure S1.** Optical absorbance data of a PET film and different CNT networks deposited onto a PET film. The purple line represents the absorbance data of a bare PET film. We prepared three different CNT networks on a bare PET film and measured optical absorbance data. First, CNTs were mixed with pure DI water with a concentration of 0.005 mg/ ml and drop casted onto a bare PET film to prepare pure CNT networks (blue line). We also mixed CNTs with Congo red solution (1mM) in DI water just like in our sensor fabrication processes (0.005 mg/ml). Here, Congo red enhanced CNT dispersion in DI water and helped preparing well-dispersed CNT solution. Then, the prepared solution was drop casted onto a PET substrate and dried to prepare CNT networks with remaining Congo red (red line). The CNT network sample was rinsed thoroughly by ethanol and N,N-Dimethylformamide for 30 min to remove Congo red dyes just like in our sensor fabrication processes (black line). The CNT network films with remaining Congo red dyes exhibited a high absorbance peak at 500nm (red line). However, the peak disappeared after the rinsing process (black line), and the rinsed CNT networks (black like) exhibited absorbance data similar to pure CNT network films (blue line). This result indicates that Congo red molecules were successfully removed from CNT networks by the rinsing during our sensor fabrication processes.

**Schematic diagram of a gas flow system for a homemade gas chamber**

_
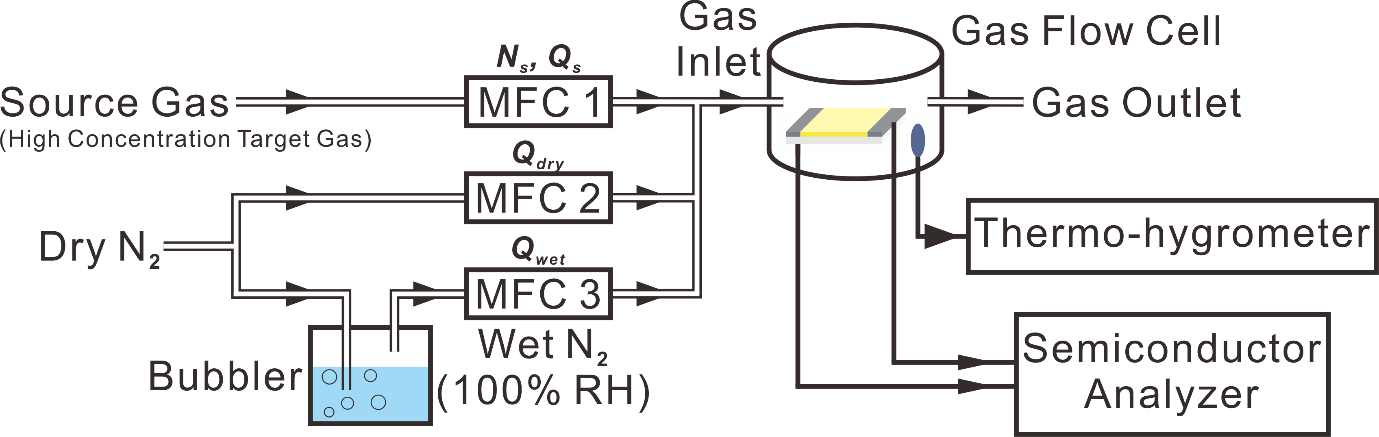
_

**Figure S2.** Schematic diagram showing our homemade gas chamber for gas sensing experiments. Here, we placed our gas sensors in a gas flow cell and passed target gas with controlled concentration and humidity into the cell using a digital mass flow controller. A test gas with desired concentrations and humidity values was prepared by mixing the source target gas (high-concentration dry SO_2_, NH_3_ and Cl_2_, purchased from RIGAS Co., Ltd in South Korea) with a mixture of dry (0% humidity) and wet (100% humidity) N_2_ gases. In this case, the concentration and humidity of target gas can be written like

$\boldsymbol{N}_{\boldsymbol{t}}=\boldsymbol{N}_{\boldsymbol{s}}\times\frac{\boldsymbol{Q}_{\boldsymbol{s}}}{\left( \boldsymbol{Q}_{\boldsymbol{dry}}+\boldsymbol{Q}_{\boldsymbol{wet}}+\boldsymbol{Q}_{\boldsymbol{s}} \right)}$ (S1)

$\boldsymbol{RH}_{\boldsymbol{t}}=\frac{\boldsymbol{Q}_{\boldsymbol{wet}}}{(\boldsymbol{Q}_{\boldsymbol{dry}}+\boldsymbol{Q}_{\boldsymbol{wet}}+\boldsymbol{Q}_{\boldsymbol{s}})}\times\mathbf{100}$ (S2)

where *N_s_*, *Q_s_*, *Q_dry_* and *Q_wet_* are the *concentration of source gas*, *the flow rate of source gas*, *the flow rate of dry N_2_ gas* and *the flow rate of wet N_2_ gas*, respectively. Using this system, we could control the relative humidity of target gas from 0% to 95% with desired gas concentration. Also, we monitored the temperature and humidity inside the gas flow cell using a thermo-hygrometer in the cell. The humidity controlling system and humidity monitoring system are independent, thus we could obtain the reliable relative humidity of target gases. As a sensor signal, the electrical conductance change of our gas sensor was measured using a semiconductor analyzer (Keithley, 4200-SCS).

**Calculation and comparison of theoretical dissociation constant of Hill equation**

The estimated value of dissociation constant *K_d-ppm_* in Figure 3 (c) is 1.82 ppm in ppm unit. To compare the estimated *K_d_* value with theoretically calculated value *K_d-theo_*, we have to express the *K_d-ppm_* value in molarity unit via ideal gas equation. However, we couldn’t directly calculate the *K_d_* value in molarity unit because we don’t know the pressure of the SO_2_ gas in N_2_ gas. Thus, we used a molarity of the N_2_ gas and a molarity ratio between SO_2_ gas and N_2_ gas. The estimated *K_d-ppm_* value of 1.82 ppm indicates that the molarity of N_2_ gas is 5.49 x 10^5^ times larger than the molarity of SO_2_ gas at equilibrium state. Thus, the physical properties such as pressure and density of applied gas are almost same with those of pure N_2_ gas. From the ideal gas law, the molarity of N_2_ gas is expressed as

$c_{N2}=\frac{n}{V}=\frac{P}{RT}$ (S3)

Here, $c_{N2}$, *n*, *V*, *P*, *R* and T are the molarity of N_2_ gas, the amount of N_2_ gas, the volume of N_2_ gas, the pressure of N_2_ gas, the ideal gas constant and the temperature of N_2_ gas, respectively. The measured value of *P* is 1.2 atm and temperature is 296 K. Thus, the *c_N2_* at equilibrium state is about 5.05 x 10^-2^ M. Using the estimated *c_N2_*, we could express the dissociation constant *K_d_* in molarity unit as 0.919 x 10^-7^ M.

To compare the dissociation constant with previously reported results, we calculated the theoretical dissociation constant *K_d-theo_* of our gas sensor via previously reported values. The SO_2_ sensing mechanism of our gas sensor is expressed as

${SO}_{2}+H_{2}O+A^{-}\rightleftarrows H^{+}+HSO_{3}^{-}+A^{-}\rightleftarrows HSO_{3}^{-}+HA$ (S4)

Here, the A^-^ and HA are yellow and red-colored methyl red, respectively. In the first process, SO_2_ gas was absorbed into water and produces bisulfite ions and H^+^ ions. In the second process, yellow-colored methyl red molecules and H^+^ ions are combined to produce red-colored methyl red molecules. In these processes, the theoretical dissociation constant of total process is written as

$\boldsymbol{K}_{\boldsymbol{d}-\boldsymbol{theo}}=\frac{\left[ {\boldsymbol{HSO}_{\boldsymbol{3}}}^{-} \right]\left[ \boldsymbol{HA} \right]}{\left[ \boldsymbol{SO}_{\boldsymbol{2}} \right]\left[ \boldsymbol{H}_{\boldsymbol{2}}\boldsymbol{O} \right]\left[ \boldsymbol{A}^{-} \right]}=\frac{\left[ {\boldsymbol{HSO}_{\boldsymbol{3}}}^{-} \right]\left[ \boldsymbol{H}^{+} \right]\left[ \boldsymbol{A}^{-} \right]}{\left[ \boldsymbol{SO}_{\boldsymbol{2}} \right]\left[ \boldsymbol{H}_{\boldsymbol{2}}\boldsymbol{O} \right]\left[ \boldsymbol{A}^{-} \right]}\times\frac{\left[ {\boldsymbol{HSO}_{\boldsymbol{3}}}^{-} \right]\left[ \boldsymbol{HA} \right]}{\left[ {\boldsymbol{HSO}_{\boldsymbol{3}}}^{-} \right]\left[ \boldsymbol{H}^{+} \right]\left[ \boldsymbol{A}^{-} \right]}=\boldsymbol{K}_{\boldsymbol{a}}\times\boldsymbol{K}_{\boldsymbol{b}}$ (S5)

Here, the *K_a_* is a dissociation constant of an acid hydrolysis reaction of SO_2_ gas. Also, the *K_b_* is dissociation constant of methyl red dye. These *K_a_* and *K_b_* values are known as 1.3 x 10^-2^ M and 7.94 x 10^-6^ M in previous papers. Thus, the theoretical dissociation constant of our gas sensor *K_d-theo_* is given by 1.03 x 10^-7^ M. The theoretical *K_d-theo_* value is similar with our experimental value of *K_d_*.

**SO_2_ detection using a methyl red-functionalized CNT-dye hybrid gas sensor under air environments**


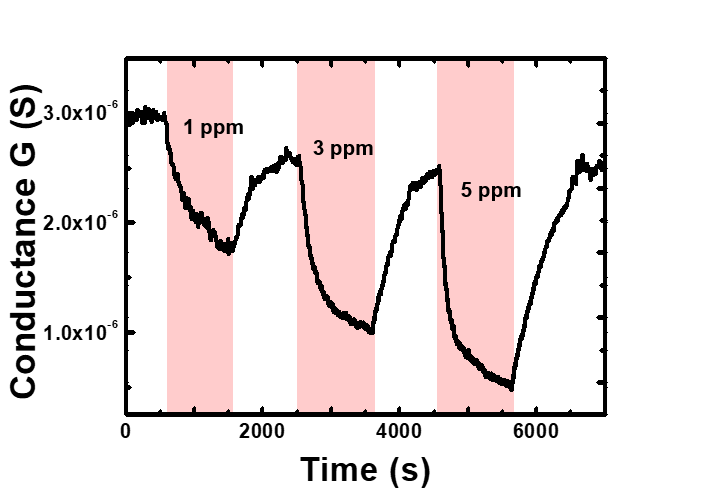


**3 ppm**

**1 ppm**

**5 ppm**

**Figure S3.** Real-time conductance measurement data obtained from a methyl red-functionalized CNT-dye hybrid gas sensor after the introduction of SO_2_ gases with different concentrations from 1 ppm to 5 ppm under *air* environments (purchased from Seoul Specialty Gases Co., composed of 21% of O_2_ and 79% of N_2_, 99.999% purity). The sensing graph was measured at 0.1 V of bias voltage and 80% of relative humidity. SO_2_ gas was applied during 1000 seconds (pink region) at intervals of about 2000 seconds.

**Selective detection via CNT-dye hybrid gas sensor**


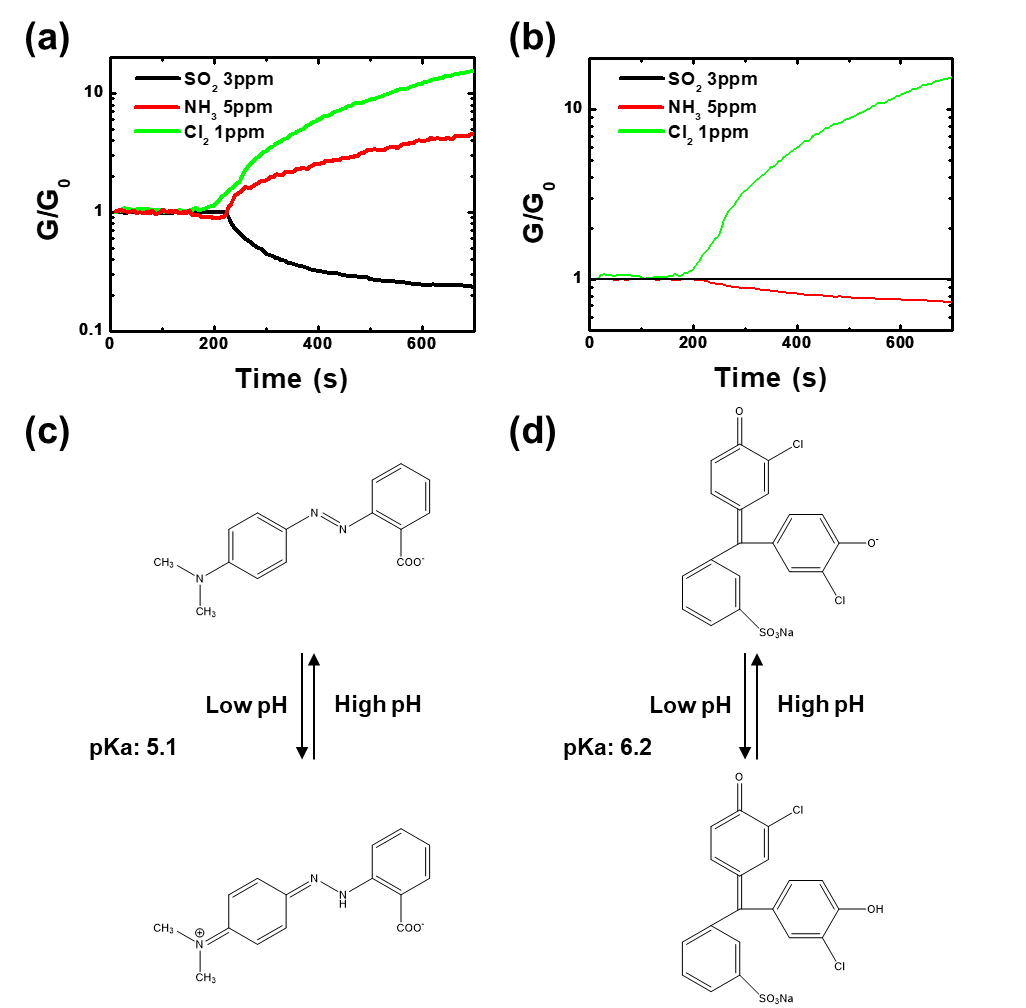


**Figure S4.** Selective detection of CNT-dye hybrid gas sensor. (a) Real-time relative conductance (*G/G_0_*) measurement data obtained from methyl red (MR) functionalized CNT-dye hybrid gas sensors after the introduction of SO_2_, NH_3_ and Cl_2_ gases. A 0.1 V of bias voltage was applied for measurements. The sensors were exposed to gases at 200 s and relative humidity was maintained during the measurements. The Henry’s raw constants of *SO_2_*, *NH_3_* and *Cl_2_* gases in water are *1.1~1.5 M/atm*, *27~76 M.atm*, and *0.062~0.095 M/atm*, respectively ^1^. (b) Real-time relative conductance (*G/G_0_*) measurement data obtained from chlorophenol red (CPR) functionalized CNT-dye hybrid gas sensors after the introduction of SO_2_, NH_3_ and Cl_2_ gases. A 0.1 V of bias voltage was applied for measurements. The sensors were exposed to gases at 200 s and relative humidity was maintained during the measurements. (c) Charge state change of methyl red induced by gas molecules (d) Charge state change of chlorophenol red induced by gas molecules.

**Response of methyl red-functionalized CNT-dye hybrid gas sensors at different relative humidity conditions.**


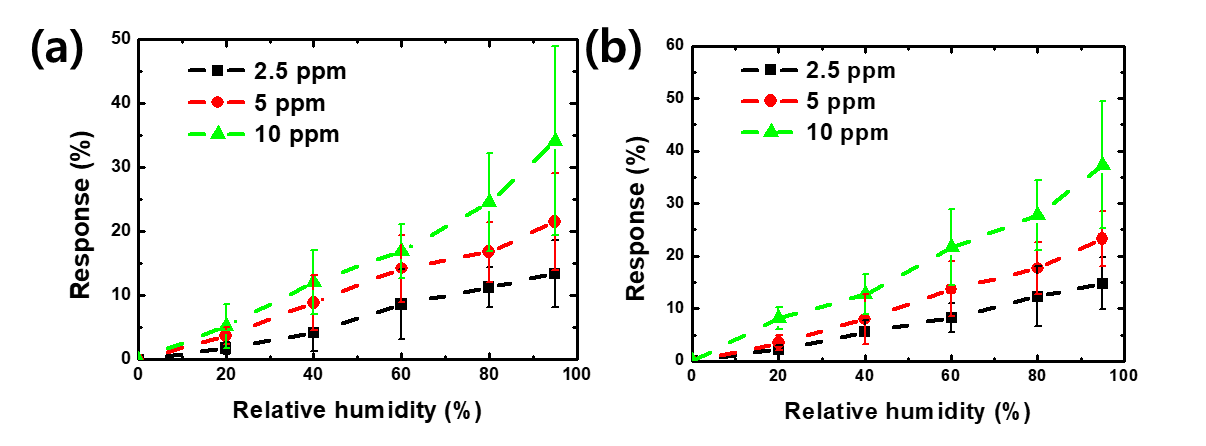


**Figure S5.** Relative humidity dependence of CNT-dye hybrid gas sensor responses to (a) NH_3_ and (b) Cl_2_ gases. The sensor responses were measured under different humidity conditions of 0%, 20%, 40%, 60%, 80%, and 95%. Each gas was tested at concentration of 2.5 ppm (black), 5 ppm (red), and 10 ppm (green).

1 Sander, R. *Compilation of Henry’s Law Constants for Inorganic and Organic Species of Potential Importance in Environmental Chemistry (Version 3)*. (1999).
